# QN-302 demonstrates opposing effects between i-motif and G-quadruplex DNA structures in the promoter of the S100P gene†

**DOI:** 10.1039/d3ob01464a

**Published:** 2023-11-06

**Authors:** Effrosyni Alexandrou, Dilek Guneri, Stephen Neidle, Zoë A. E. Waller

**Affiliations:** a School of Pharmacy, University College London 29-39 Brunswick Square London WC1N 1AX UK s.neidle@ucl.ac.uk z.waller@ucl.ac.uk

## Abstract

GC-rich sequences can fold into G-quadruplexes and i-motifs and are known to control gene expression in many organisms. The potent G-quadruplex experimental anticancer drug QN-302 down-regulates a number of cancer-related genes, in particular *S100P*. Here we show this ligand has strong opposing effects with i-motif DNA structures and is one of the most potent i-motif destabilising agents reported to date. QN-302 down-regulates the expression of numerous cancer-related genes by pan-quadruplex targeting. QN-302 exhibits exceptional combined synergistic effects compared to many other G-quadruplex and i-motif interacting compounds. This work further emphasises the importance of considering G-quadruplex and i-motif DNA structures as one dynamic system.

## Abstract 2

The potent G-quadruplex experimental drug QN-302 has strong opposing effects with i-motif DNA structures at the promoter of *S100P*.

## Graphical abstract

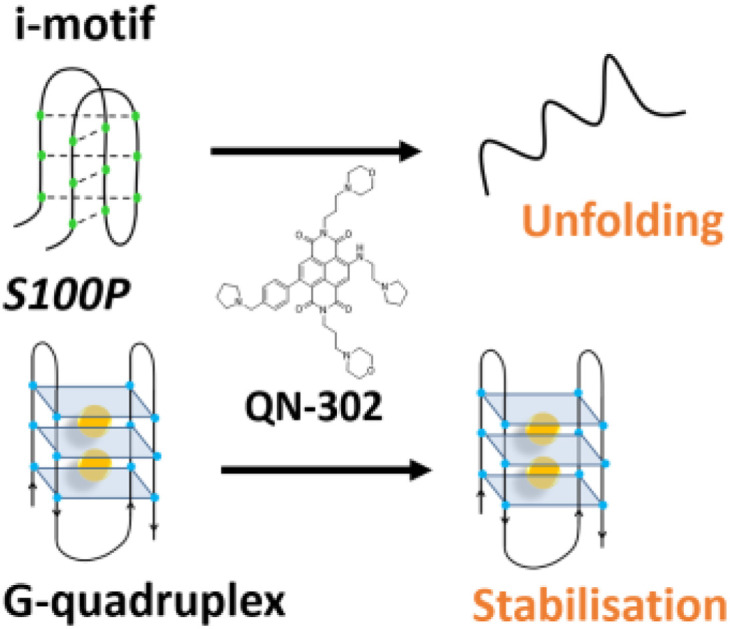

Promoter sequences within cancer-related genes frequently contain repeats of short G-tracts that can fold into higher-order quadruplex structures under appropriate conditions.^1–5^ Their complementary C-tract strands can also fold into i-motif arrangements.^6,7^ Stabilization of these structures by appropriate small-molecule compounds can result in transcriptional inhibition, and ultimately to cancer cell death.^1,5^ Several thousand such compounds have been described, and some show promise as potential drug candidates.^8–12^ We have developed several series of substituted naphthalene diimide derivatives,^13–15^ and the most recent, QN-302 (Fig. 1), shows high potency in cell growth inhibition assays, favourable pharmacological properties and antitumour activity in several *in vivo* cancer models.^16^ The transcriptional profile in cancer cells of genes down-regulated by QN-302 is in accordance with the hypothesis that it is a pan-quadruplex stabilising agent, affecting genes in several important cancer-related pathways.^16^ However, to date it cannot be excluded that QN-302 also stabilises i-motif structures formed on the complementary C-rich strand of G-quadruplex sequences. The present study addresses this issue with a major gene target as an exemplar.

**Fig. 1:**
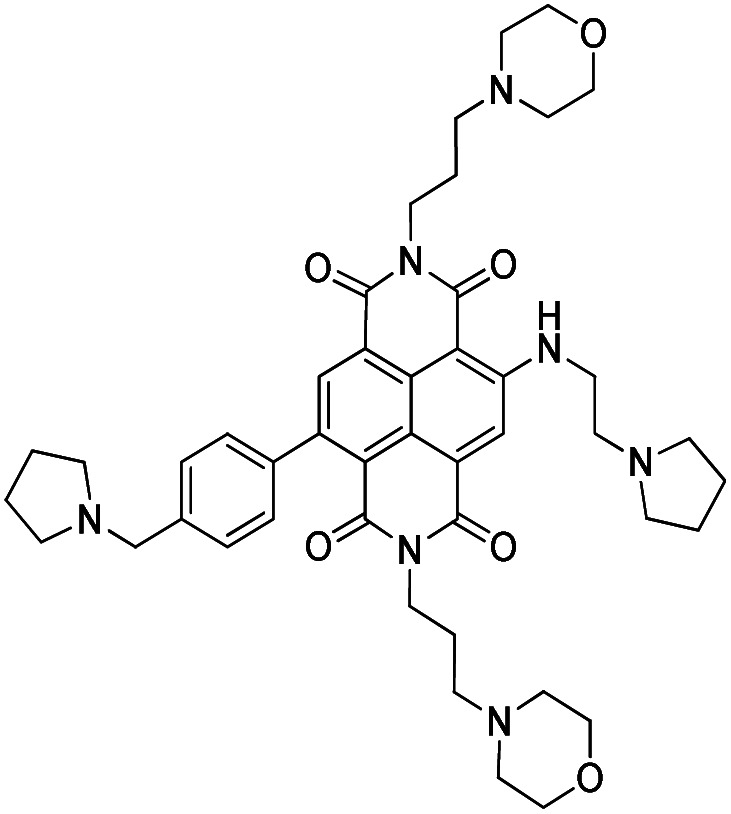
Structure of QN-302.

QN-302 is currently in clinical development with Qualigen Therapeutics Inc. It has been granted Orphan Drug Designation status for the treatment of pancreatic cancer, clearance has been granted by the FDA in the USA to proceed to clinical trials for human cancers, which are now underway.

QN-302 down-regulates the expression of a number of significant cancer-related genes,^16^ including the *S100P* gene in cancer cells and in a xenograft model of pancreatic cancer.^17^ This gene codes for a small (10.4 kDa) calcium-binding protein and is highly upregulated in 70% of human pancreatic cancer patients, correlating with disease status.^18,19^ The S100P protein has been proposed as a plausible biomarker for diagnostic purposes and as a therapeutic target in pancreatic cancer.^20,21^ The *S100P* promoter^22^ contains a C-rich sequence containing four C-tracts on the coding strand, 48 nucleotides upstream from the transcription start site. The complementary four G-tract sequence on the template strand forms a stable G-quadruplex, which is further stabilised by QN-302.^17^ Here we report on the biophysical characterisation and comparison of both the G-rich and C-rich sequences from the promoter region of *S100P*.

We initially characterised both the C-rich [5′-TCCCAACCCCACTGTCCCACCCT-3′] and G-rich [5′- AGGGTGGGACAGTGGGGTTGGGA-3′] sequences from the promoter region of the *S100P* gene. All experiments were performed in 10 mM lithium cacodylate and 100 mM KCl. The G-quadruplex forming sequence was examined at pH 7.0 and the i-motif at between pHs 4.0 and 8.0. UV melting and annealing experiments showed that the G-rich sequence had a *T*_m_ of 75.0 ± 0.2 °C and a *T*_a_ of 73.3 ± 0.7 °C at pH 7.0 (Fig. S1†). This is consistent with our previous CD experiments indicating that the G-quadruplex structure formed would be highly stable under physiological conditions.^17^ The complementary C-rich sequence had a *T*_m_ of 45.4 ± 0.6 °C and a *T*_a_ of 43.0 ± 0.0 °C at pH 5.5 (Fig. S2†). This *T*_m_ is similar to the melting temperature of other i-motifs of this length with three-cytosine long tracks at the same pH.^23^ UV thermal difference spectroscopy on the C-rich sequence showed positive peaks at 240 and 265 nm and a negative peak at 295 nm (Fig. 2, left), consistent with i-motif structure.^24^ Circular dichroism studies at acidic pH gave a spectrum with a positive peak at 288 nm and a negative peak at 260 nm, which is also consistent with an i-motif structure^25^ (Fig. 2, right). The i-motif forming sequence was found to have a transitional pH (pH_T_) of 6.4, which indicates that this C-rich sequence can form an i-motif at near-neutral pH.^23,26^

**Fig. 2:**
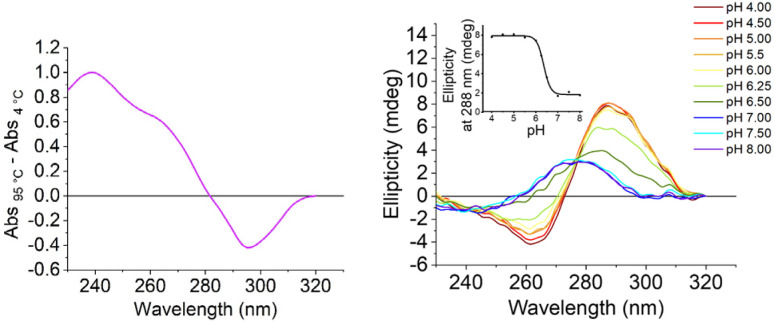
Left: thermal difference spectra of the C-rich i-motif forming *S100P* sequence: at 2.5 μM in 10 mM lithium cacodylate and 100 mM KCl buffer at pH 5.5. Right: CD spectra of the C-rich *S100P* sequence at 10 μM DNA in 10 mM lithium cacodylate and 100 mM KCl buffer at pH as indicated. Inset: corresponding plot of ellipticity at 288 nm at the different pHs to determine the transitional pH.

QN-302 is one of the most potent G-quadruplex binding ligands reported to date with a *K*_d_ of 4.9 nM for the G-quadruplex forming sequence from hTERT.^15^ It was previously shown to stabilise the G-quadruplex from the promoter region of *S100P* with Δ*T*_m_ values of 7.4 ± 0.2 °C at 10 μM (1 eq.), 17.0 ± 0.1 °C at 20 μM (2 eq.) and 20.0 ± 1.3 °C at 50 μM (5 eq.) (Table 1).^17^ These data indicates that QN-302 has a strong stabilising effect on the G-quadruplex structure formed.

**Table 1:** Change in melting temperature (Δ*T*_m)_ of the *S100P* G-quadruplex and i-motif with QN-302 measured by CD melting experiments

[QN-302] μM	Δ*T*_m_ (°C)	Δ*T*_m_ (°C)
	*S100P* G – quadruplex^17^	*S100P* i – motif
10	7.4 ± 0.2	−6.5 ± 1.7
20	17.0 ± 0.1	−14.3 ± 0.1
50	20.0 ± 1.3	−20.7 ± 1.1

We then focused in detail on the effects of QN-302 on the C-rich sequence from the *S100P* promoter. Δ*T*_m_ values in the presence of QN-302 were determined in 10 mM lithium cacodylate, 100 mM KCl at pH 5.5, where the *S100P* sequence would be fully folded (Fig. 2). At 10 μM (1 eq.) of QN-302 the Δ*T*_m_ values were found to be −6.5 ± 1.7 °C, −14.3 ± 0.1 °C at 20 μM (2 eq.) and −20.7 ± 1.1 °C at 50 μM (5 eq.), demonstrating a dose-dependent destabilisation of i-motif structure by QN-302 (Fig. 3, Table 1 and Fig. S4†). Other known G-quadruplex ligands such as berberine, BRACO-19, Phen-DC3, pyridostatin, RHPS4 and TmPyP4 have previously been shown to destabilise i-motifs, but to a lesser extent.^27–29^ For example, BRACO-19 has a Δ*T*_m_ values of −7.3 ± 0.7 °C for the i-motif forming sequence from the promoter region of the DAP gene^28^ and −13.4 ± 0.5 °C for the i-motif from the human telomere. These Δ*T*_m_ values are significantly smaller compared to our observations with QN-302. Di Porzio, Galli *et al.* have synthesised bis-triazolyl-pyridine derivatives that appear to have highly destabilising effects on the *c-Myc* and the hTelo i-motifs with Δ*T*_m_ values of up to −29 ± 1 °C in one case. However, this destabilisation was achieved with double the number of ligand equivalents (10 molar equivalents) in phosphate buffer at pH 5.0.^29^ These ligands did not have the same high stabilising effect on their respective G-quadruplexes as we observe with QN-302. To the best of our knowledge QN-302 is one of the most potent destabilising agents for i-motifs reported to date. Highly destabilising activity was also observed when QN-302 was tested against the i-motif forming sequences from the human genome including the telomeric sequence (hTelo), the insulin linked polymorphic region (ILPR) and the promoter region of DAP (Fig. S5–S7 and Table S1†).

**Fig. 3:**
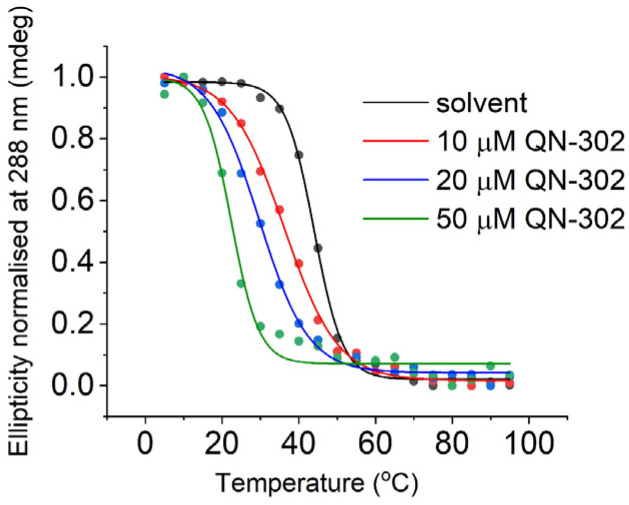
Representative CD melting experiments with 10 μM *S100P* i-motif in 10 mM lithium cacodylate 100 mM KCl buffer (pH 5.5), and 0, 10, 20 or 50 μM QN-302, as indicated.

To further investigate the destabilising effects of the *S100P* i-motif by QN-302 CD titrations were performed (Fig. 4). Upon addition of QN-302 at a concentration range from 0 to 110 μM, the CD signal intensity at 288 nm was found to decrease in a dose-dependent fashion until a point at ∼50 μM beyond which no further reduction in the ellipticity was observed. The decrease in the CD signal suggested ligand-dependent disruption of the *S100P* i-motif, consistent with unfolding of the structure to a single strand. This agrees with the CD melting experiments showing destabilisation. A plot of ellipticity against QN-302 concentration gave a sigmoidal-shaped curve (Fig. 4), indicative of a cooperative unfolding effect. By fitting the sigmoidal-shaped curves to the Hill 1 equation using Origin software, we obtained Hill coefficients (*n*) of 2.3 ± 0.2. This reveals that the binding of QN-302 exhibits positive cooperativity (*n* > 1) for the *S100P* i-motif. Additionally, the concentration of QN-302 that is required to reach 50% reduction of the molar ellipticity was determined to be 31 ± 4 μM. Analogous CD titration experiments with the G-quadruplex forming sequence showed no significant changes in topology (Fig. S3†). Indicating that the ligand is G-quadruplex stabilising and has both i-motif destabilising and unfolding properties. Taken together, the biophysical data illustrate the dynamic interplay of the two higher order DNA structures.

**Fig. 4:**
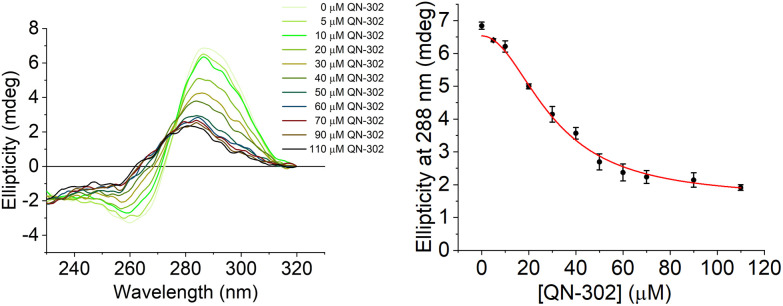
Left: CD titration of the C-rich *S100P* sequence (10 μM) and QN-302 (0–110 μM) in 10 mM lithium cacodylate and 100 mM KCl buffer at pH 5.5. Right: plot of ellipticity at 288 nm against QN-302 concentration and corresponding Hill 1 fitting.

To further compare the affinity of QN-302 for the *S100P* i-motif and G-quadruplexes, UV titrations were performed (Fig. S8–S13 and Table S2†) and the dissociation constants (*K*_d_) were determined. The *K*_d_ for the G-quadruplex (*K*_d_ = 2.0 ± 0.3 μM) was found to be about six times lower than for the i-motif (*K*_d_ = 11.7 ± 2.9 μM), indicating that QN-302 has higher affinity for G-quadruplex compared to i-motif. This was not unexpected, given QN-302 was designed to target G-quadruplex structures.

Numerous studies have shown that high expression of the *S100P* gene is correlated with pancreatic cancer progression in humans.^18–21^ The proposed mode of action involves the stabilisation of the G-quadruplex sequence in the promoter.^17,22^ This stabilisation would inhibit transcription factor binding and the progression of RNA polymerase, resulting in direct downregulation of *S100P* gene expression at the transcriptional level analogous to other ligands such as pyridostatin.^16,17,30^ In this study we have further examined the mechanistic details of QN-302 interacting with the higher-order structures that can be formed in this promoter region of the *S100P* gene and in particular have examined the potential role of the i-motif formed by the C-rich strand. QN-302 has a strong destabilising effect on the *S100P* i-motif as it is illustrated by CD melting and titration experiments. Therefore, QN-302 by stabilizing the G-quadruplex structure and destabilizing the i-motif structure, has a dual role and may exert a synergistic effect on the inhibition of transcription of the *S100P* gene. Ligand-induced G-quadruplex stabilization inhibits gene expression whereas stabilization of i-motifs could activate transcription.^31,32^ This highlights the importance of evaluating the effects of a compound on both the i-motifs and the G-quadruplexes potentially formed from a duplex region of appropriate sequence. This is particularly important given the fact that G-quadruplex and i-motif formation in cells are interdependent.^33^ In the case of *S100P*, there is biological evidence that QN-302 can switch off gene expression^17^ which may be a consequence of both the stabilisation of the G-quadruplex and the destabilisation of the i-motif. This suggests how these two alternative structures operate together in the *S100P* promoter.

The findings reported here demonstrate that QN-302 both strongly stabilizes the *S100P* promoter G-quadruplex and strongly destabilizes the complementary i-motif *in vitro*. These data are consistent with and supportive of previous conclusions^16^ that QN-302 down-regulates the expression of numerous cancer-related genes by pan-quadruplex targeting. This is particularly important given the recent analysis of TCGA PanCancer Atlas PDAC datasets that indicate poor prognosis in patients with high *S100P* expression.^34^ QN-302 exhibits exceptional combined synergistic effects compared to many other G-quadruplex and i-motif interacting compounds. Overall, this work further emphasises the importance of considering these two alternative DNA structures as one dynamic system and as one target.

## Data availability

Data is available on Figshare: 10.6084/m9.figshare.24476551.

## Author contributions

S. N. and Z. A. E. W. conceived the study, E. A, D. G. and Z. A. E. W. designed the experiments E. A. and D. G. performed the experiments E. A., D. G., S. N. and Z. A. E. W. wrote the paper, contributed to the manuscript revision, and approved the final version.

## Conflicts of interest

S. Neidle is a paid consultant and Advisory Board member of Qualigen Inc.

## Supplementary Material

OB-022-D3OB01464A-s001
