# Supplementary material for: QN-302 demonstrates opposing effects between i-motif and G-quadruplex DNA structures in the promoter of the S100P gene
Source: Org Biomol Chem. 2023 Nov 6;22(1):55–8. doi: 10.1039/d3ob01464a (PMC10732280; doi:10.1039/d3ob01464a)
Supplement: OB-022-D3OB01464A-s001 [file OB-022-D3OB01464A-s001.pdf]

Electronic Supplementary Material (ESI) for Organic & Biomolecular Chemistry.
This journal is © The Royal Society of Chemistry 2023

SUPPLEMENTARY INFORMATION

QN-302 demonstrates opposing effects between i-motif and G-quadruplex DNA structures in the promoter of the S100P gene

Effrosyni Alexandrou, Dilek Guneri, Stephen Neidle, Zoë A. E. Waller*

School of Pharmacy, University College London,
29-39 Brunswick Square, London, WC1N 1AX, UK

METHODS

Oligonucleotides

The following putative G-quadruplex and i-motif forming sequences from the *S100P* promoter and the i-motif forming sequences of *DAP*, *hTelo* and *ILPR* were purchased from Eurogentec UK and were supplied reverse phase HPLC purified, as a dry solid:

S100P G-quadruplex: 5'-d[AGGGTGGGACAGTGGGGTTGGGA]-3'

S100P i-motif: 5'-d[TCCCAACCCCACTGTCCCACCCT]-3'

DAP i-motif: 5'-d[CCCCCGCCCCCGCCCCCGCCCCCGCCCCCG]-3'

hTelo i-motif: 5'-d[TAACCCTAACCCTAACCCTAACCC]-3'

ILPR i-motif: 5'-d[TGTCCCCACACCCCTGTCCCCACACCCCTGT]-3'

The DNA was initially dissolved as a stock solution in purified water at approximately 1 mM, concentrations were confirmed using a Nanodrop and further dilutions were carried out in the respective buffer. Samples were thermally annealed in a heat block at 95 °C for 5 minutes and cooled slowly to room temperature overnight. Stock solutions of QN-302 were prepared in buffer, with some drops of 0.1 M HCl to aid solubilization. Data were analysed using the OriginLab package (https://www.originlab.com/).

UV Melting/Annealing, Thermal Difference Spectroscopy (TDS) and UV titrations

UV melting/annealing and TDS experiments were performed using the Jasco V-750 UV–Vis spectrometer. The C-rich *S100P* sequence was annealed at 2.5 μM in 10 mM lithium

cacodylate and 100 mM KCl buffer at pH 5.5 while 5 µM G-rich *S100P* samples were annealed in 10 mM lithium cacodylate and 100 mM KCl buffer at pH 7.0. After annealing, the samples (250 µL) were transferred to quartz 10 mm cuvettes and stoppered to reduce evaporation.

For the UV melting/annealing experiments, the absorbance of the samples was recorded at every 1 °C increase/decrease in at least two cycles at 295 nm and 260 nm. Initially, the samples were held at 4 °C for 10 min followed by gradual increase to 95 °C at a rate of 0.5 °C/min (melting). When the temperature reached 95 °C, it was held for 10 min before the process was reversed (annealing). The average melting (T_m) and annealing temperature (T_a) were identified by the first derivative method for each measured cycle.

The thermal difference spectra (TDS) of the *S100P* i-motif was obtained by measuring the absorbance spectrum from 230 nm to 320 nm at 4 °C for the folded DNA structure and at 95 °C for the unfolded structure. The sample was equilibrated for 5 to 10 minutes at each of the two temperatures before recording the absorbance. The TDS signature was determined by subtracting the absorbance spectra of the folded structure from the unfolded structure, zero corrected at 320 nm, and then normalised to the maximum absorbance.

For the UV titrations, DNA samples were annealed at 200 µM in 10 mM lithium cacodylate and 100 mM KCl buffer at pH 5.5 for the C-rich *S100P* sequence and pH 7 for the G-rich *S100P* sequence. Firstly, 250 µL of 5 µM QN-302 ligand in 10 mM lithium cacodylate and 100 mM KCl buffer at pH 5.5 were placed in quartz 10 mm cuvette. During the titration, an aliquot of annealed 200 µM DNA solution was added to the cuvette and the sample scanned in wavelength range of 425 – 580 nm, data interval 0.5 nm and the temperature 20 °C. Two different methods were employed for calculating the fraction bound. The first method was based on recording the change in absorbance at the wavelength of maximum absorbance before addition of DNA (520 nm). The second method was based on recording the change in wavelength at the maximum absorbance after every addition of the DNA. In both cases the data was then converted to fraction bound where 0 indicates unbound (no DNA added) and 1 fully bound (when the change in the spectrum had plateaued). The fraction bound was then plotted against the concentration of the DNA in the sample and these data was fitted to the Rectangular Hyperbola Function according to equation 1.[1]

$$y = \frac{nk_a x}{1 + k_a x} \; (eq.\,1)$$

Where y = the bound fraction x = concentration of the DNA, n = stoichiometry and k_a is the association constant. The dissociation constant (k_d) was calculated using equation 2. Final values are given as the average and standard deviation of two repeats.

$$k_d = \frac{1}{k_a} \; (eq.\,2)$$

Circular Dichroism (CD) Spectroscopy

The CD spectra of the *S100P* C-rich sequence at different pH values, were recorded on a Jasco J-1500 spectropolarimeter using a 1 mm path length quartz cuvette under a constant flow of nitrogen. The samples were diluted to 10 µM in 10 mM lithium cacodylate and 100 mM KCl buffer at pH values ranging from 4.0 to 8.0 and annealed as described above (100 µL per sample). Four spectra scans were accumulated ranging from 200 nm to 320 nm for the buffer at each pH (blank) and DNA samples and measured at 20 °C with a data pitch at 0.5 nm, scanning speed of 200 nm/min with 1 second response time, 1 nm bandwidth, and 200 mdeg sensitivity. Data was zero corrected at 320 nm and transitional pH (pH_T) was determined from the inflection point of the Boltzmann sigmoidal fit for the measured ellipticity at 288 nm and pH range.

Circular dichroism spectroscopy was used to measure any ligand-induced effects on *S100P* the i-motif and G-quadruplex DNA structure. Scans were accumulated ranging from 230 nm to 320 nm. DNA samples (10 µM) were thermally annealed in 10 mM lithium cacodylate, 100 mM KCl pH 7 (for the G-quadruplex) and pH 5.5 (for the i-motif) at 100 µL final sample volume. QN-302 was titrated step-wise via consecutive additions of ligand at the following concentration ranges: 0 – 100 µM QN-302 for the G-quadruplex and 0 – 110 µM QN302 for the i-motif. Titrations presented are the average of two repeats and were corrected for the solvent effect, smoothed using the Savitzki-Golay method at 10 points of window and then zero corrected at 320 nm. Analysis and processing of the data was performed using OriginLab data analysis software to plot the normalised ellipticity at 288 nm for the i-motif against ligand concentration. The sigmoidal curves of the ellipticity at 288 nm against ligand concentration

were fitted to the Hill 1 equation to obtain the Hill coefficients (n) and the concentrations of ligand that are required to reach 50% reduction of the molar ellipticity ($[D]_{50\%}$).

Melting experiments for the *S100P* sequences were performed in the presence and absence of 1, 2 and 5 ligand equivalents (10 µM, 20 µM and 50 µM QN-302).Melting experiments for the *DAP, hTelo and ILPR* sequences were performed in the presence and absence of 5 ligand equivalents (50 µM QN-302).Samples were heated at a rate of 1 °C/min from 5 to 95 °C and measuring at 5 °C intervals. The temperature at which 50% of the thermal denaturation had taken place (T_m) was calculated by using OriginLab data analysis software to plot normalised ellipticity against temperature. These data were fitted with sigmoidal curves, to determine the T_m values. Final values are given as the average and standard deviation of two repeats.

SUPPLEMENTARY DATA

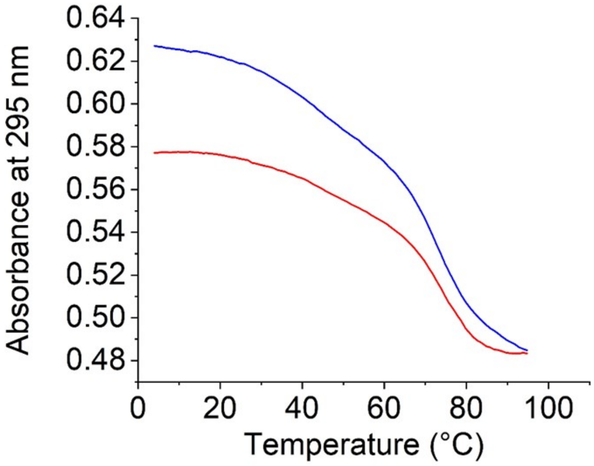

Figure S1. UV melting (red) and annealing (blue) of the G-rich *S100P* sequence: 5'- AGGGTGGGACAGTGGGGTTGGGA-3' at 295 nm of 5 µM DNA in 10 mM lithium cacodylate and 100 mM KCl buffer at pH 7.

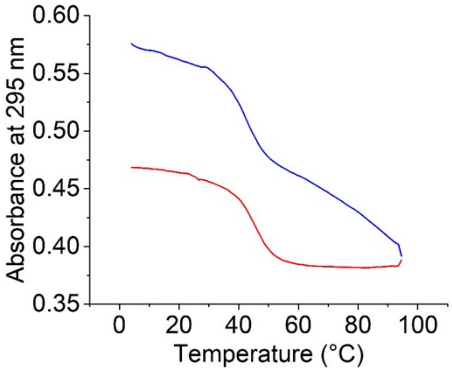

Figure S2. UV melting (red) and annealing (blue) of the C-rich *S100P* sequence: 5'-TCCCAACCCCACTGTCCCACCCT-3' at 295 nm of 2.5 µM DNA in 10 mM lithium cacodylate and 100 mM KCl buffer at pH 5.5.

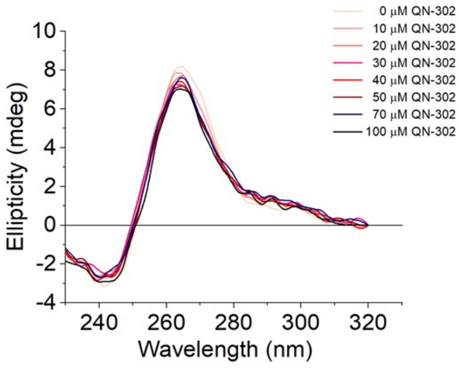

Figure S3 CD titration of the G-rich G-quadruplex forming *S100P* sequence: 5'- AGGGTGGGACAGTGGGGTTGGGA-3' (10 µM) and QN-302 (0 – 100 µM) in 10 mM lithium cacodylate and 100 mM KCl buffer at pH 7.

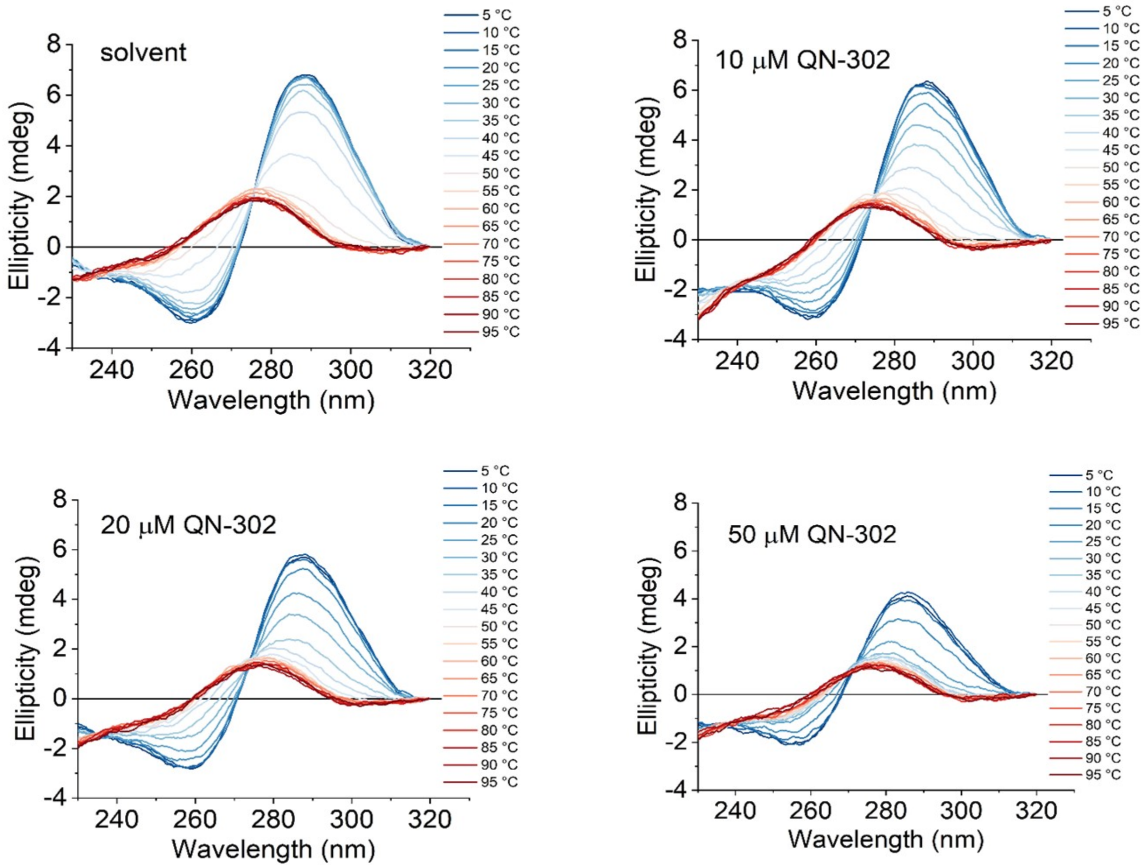

Figure S4. Examples of CD melts of the C-rich *S100P* sequence: 5'- TCCCAACCCCACTGTCCCACCCT -3' (10 µM) and QN302 (0 – 50 µM) in 10 mM lithium cacodylate and 100 mM KCl buffer at pH 5.5.

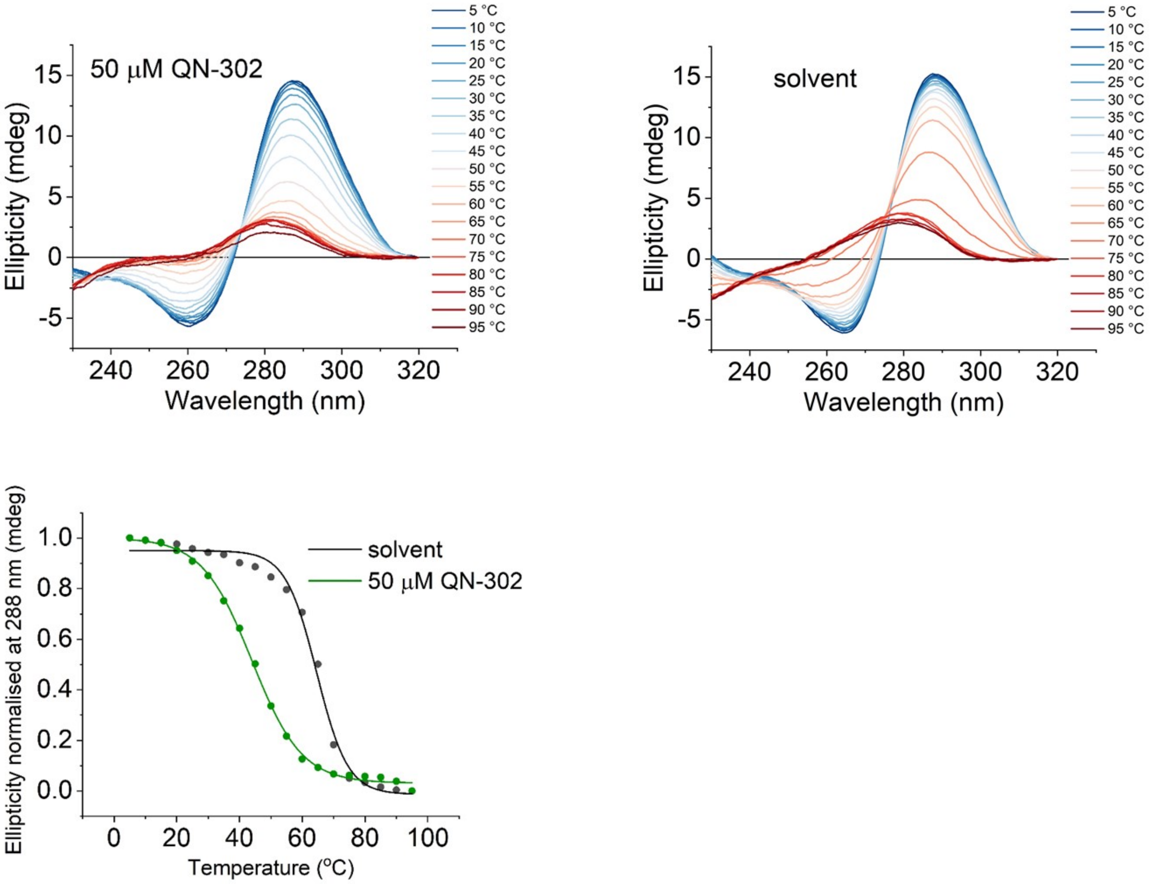

Figure S5. Representative CD melting experiments of the C-rich *DAP* sequence: 5′-CCCCCGCCCCCGCCCCCGCCCCCGCCCCCG-3′ (10 μM) and QN302 (0 and 50 μM) in 10 mM lithium cacodylate and 100 mM KCl buffer at pH 5.5.

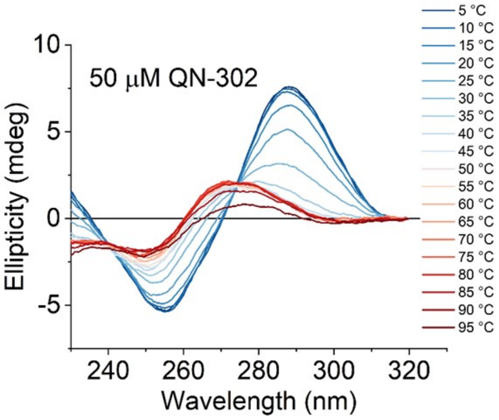

Figure S6. Representative CD melting experiments of the C-rich *htelo* sequence: 5'-TAACCCTAACCCTAACCC TAACCC -3' (10 μM) and QN302 (0 and 50 μM) in 10 mM lithium cacodylate and 100 mM KCl buffer at pH 5.5.

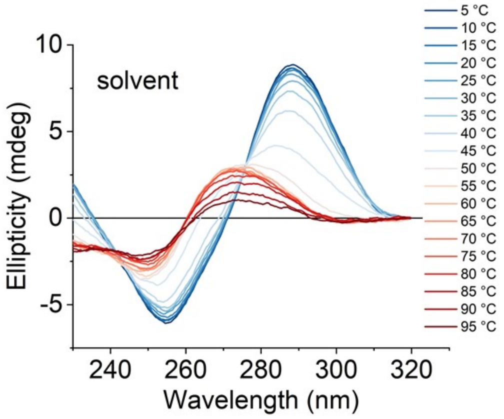

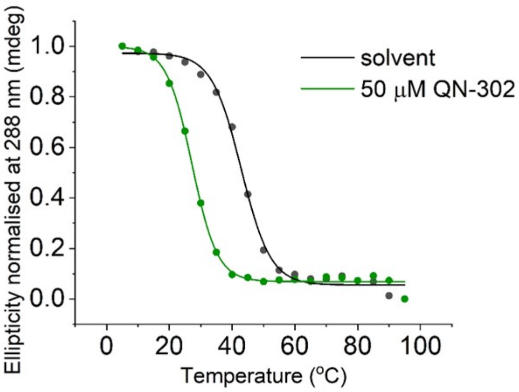

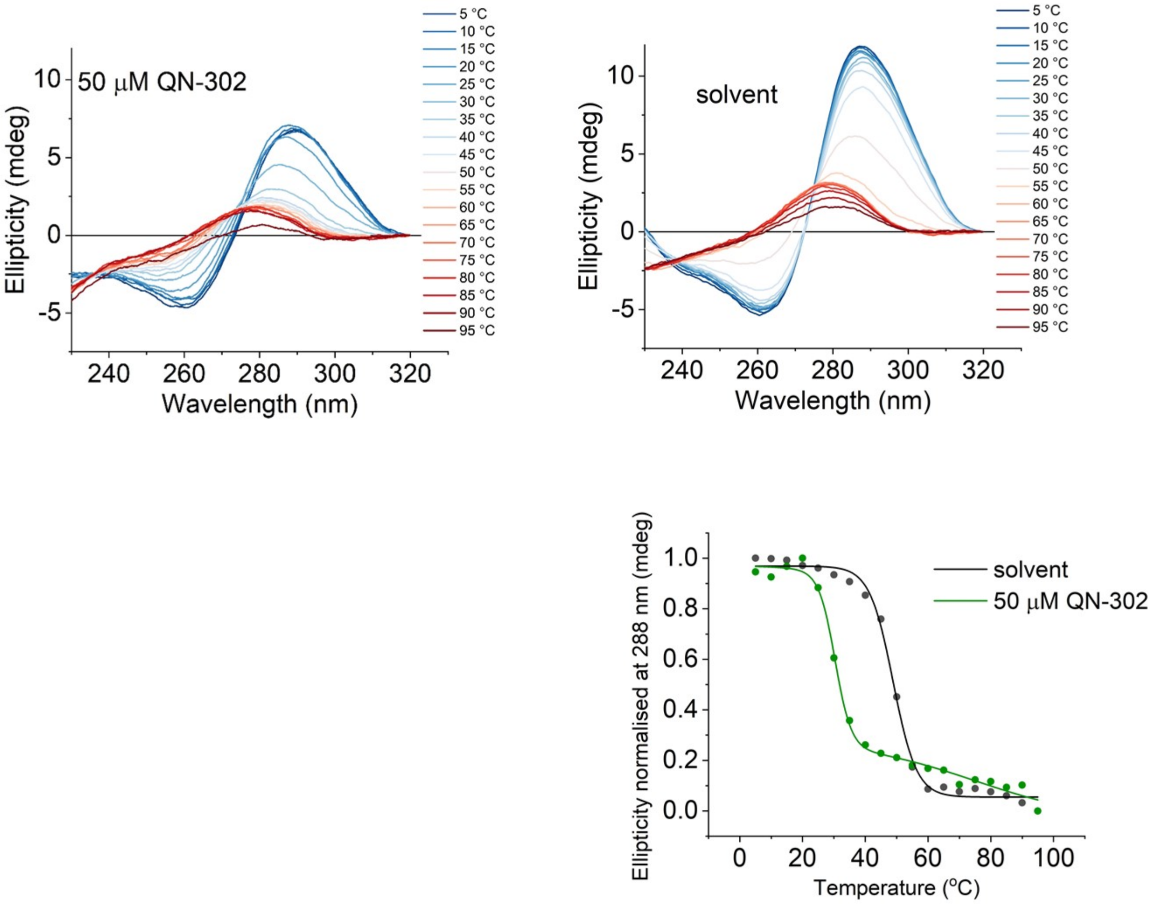

Figure S7. Representative CD melting experiments of the C-rich *ILPR* sequence: 5’-TGTCCCCACACCCCTGTCCCCACACCCCTGT-3’ (10 µM) and QN302 (0 and 50 µM) in 10 mM lithium cacodylate and 100 mM KCl buffer at pH 5.5.

Table S1. Change in melting temperature (ΔT_m) of the *DAP, hTelo* and *ILPR* i-motif with QN-302 measured by CD melting experiments.

**[QN-302] µM**	**ΔT_m (°C) *DAP* i-motif**	**ΔT_m (°C) *hTelo* i-motif**	**ΔT_m (°C) *ILPR* i-motif**
50	-21.6 ± 1.1	-16.6 ± 0.7	-18.8 ± 0.4

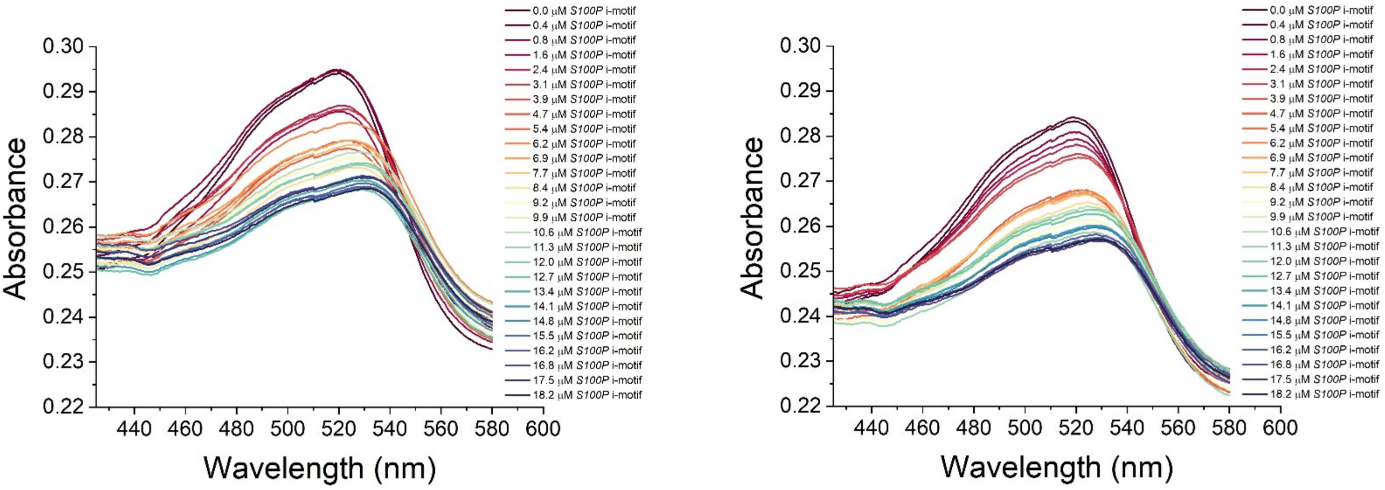

Figure S8. UV titration spectra. UV titration of QN-302 with *S100P* i-motif. Conditions: [QN-302] = 5 µM in solution buffer (10 mM Lithium cacodylate, pH 5.5 and 100 mM KCl); titrant: [*S100P* i-motif] = 0−18.2 µM in solution buffer (10 mM Lithium cacodylate, pH 5.5 and 100 mM KCl).

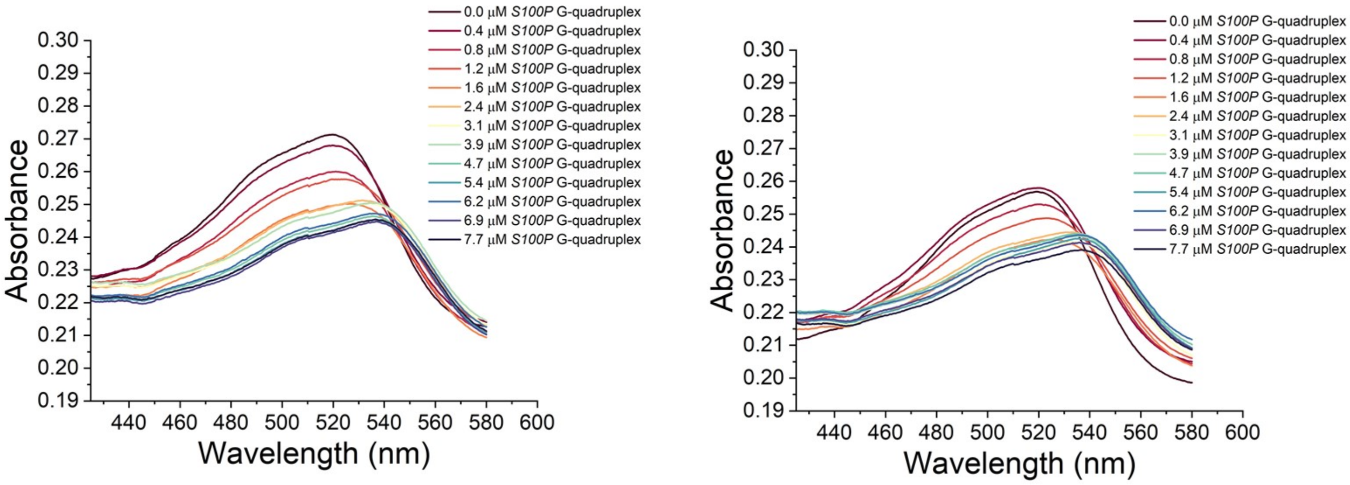

Figure S9. UV titration spectra. UV titration of QN-302 with *S100P* G-quadruplex. Conditions: [QN-302] = 5 µM in solution buffer (10 mM Lithium cacodylate, pH 5.5 and 100 mM KCl); titrant: [*S100P* G-quadruplex] = 0−7.7 µM in solution buffer (10 mM Lithium cacodylate, pH 7 and 100 mM KCl).

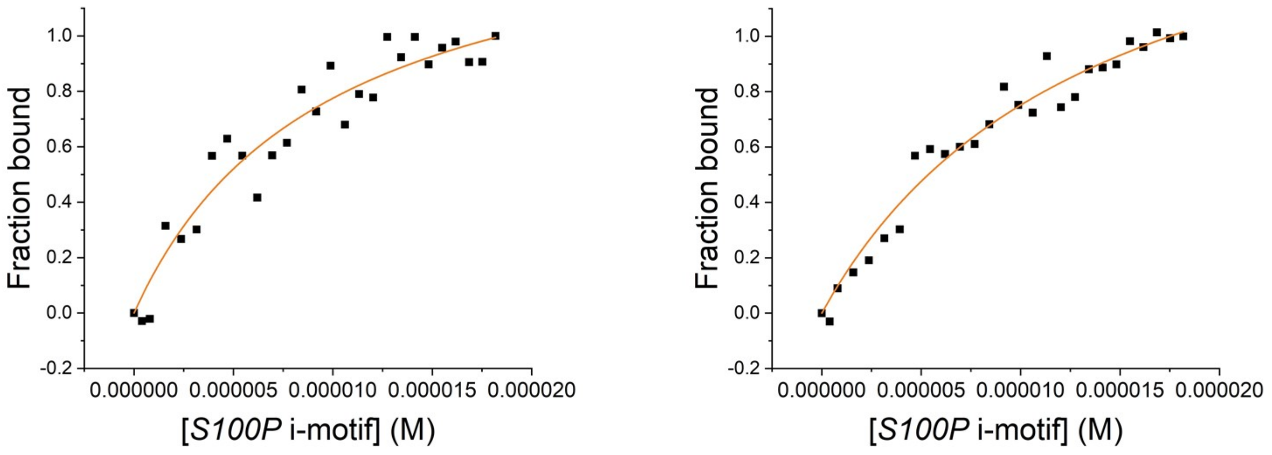

Figure S10. UV titration of QN-302 with *S100P* i-motif. Fraction bound was calculated based on the change in absorbance at the wavelength of maximum absorbance when the ligand is unbound – 519.5 nm (method 1).

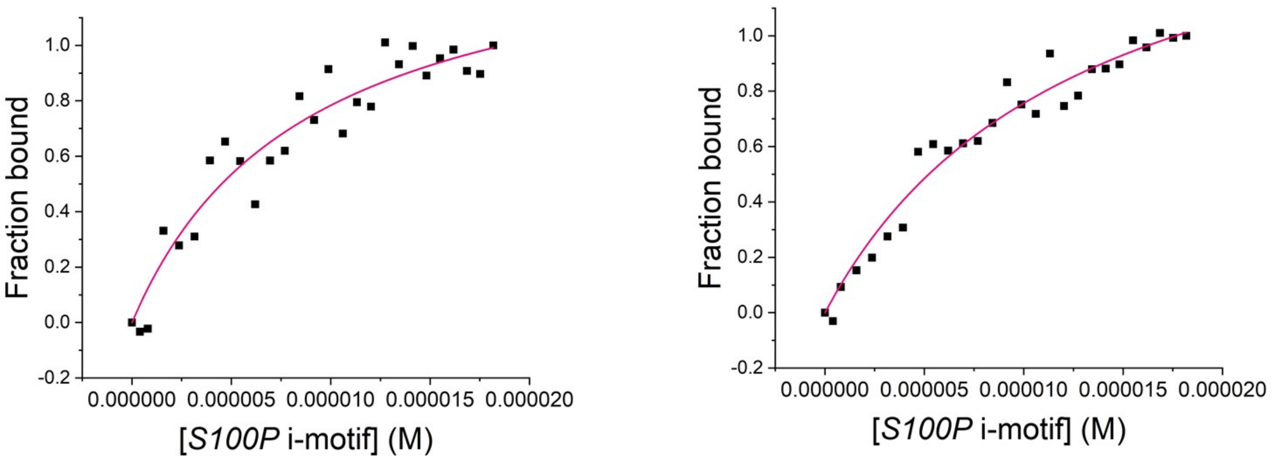

Figure S11. UV titration of QN-302 with *S100P* i-motif. Fraction bound was calculated based on the change in the maximum absorbance after every addition of the DNA (method 2).

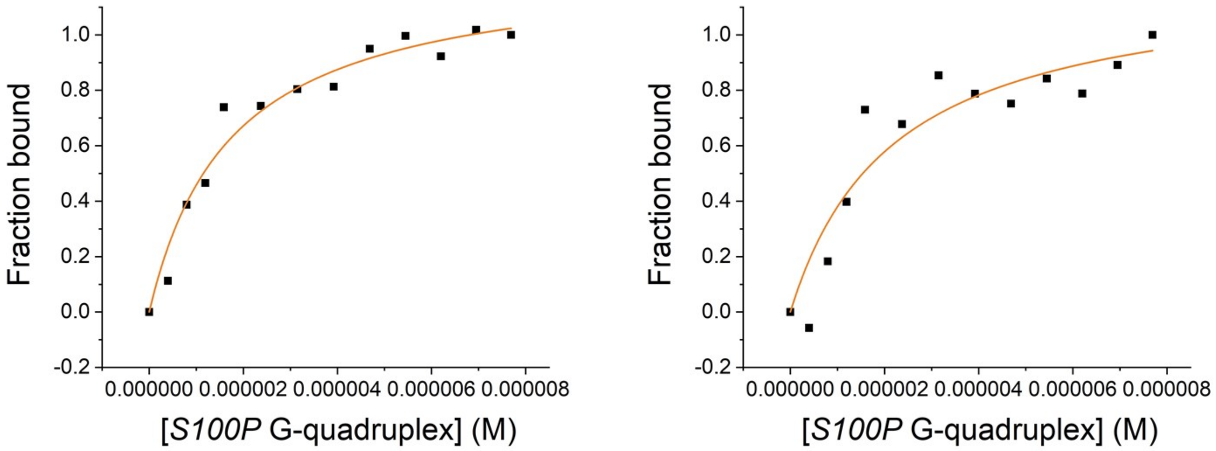

Figure S12. UV titration of QN-302 with *S100P* G-quadruplex. Fraction bound was calculated based on the change in absorbance at the wavelength of maximum absorbance when the ligand is unbound – 520 nm (method 1)

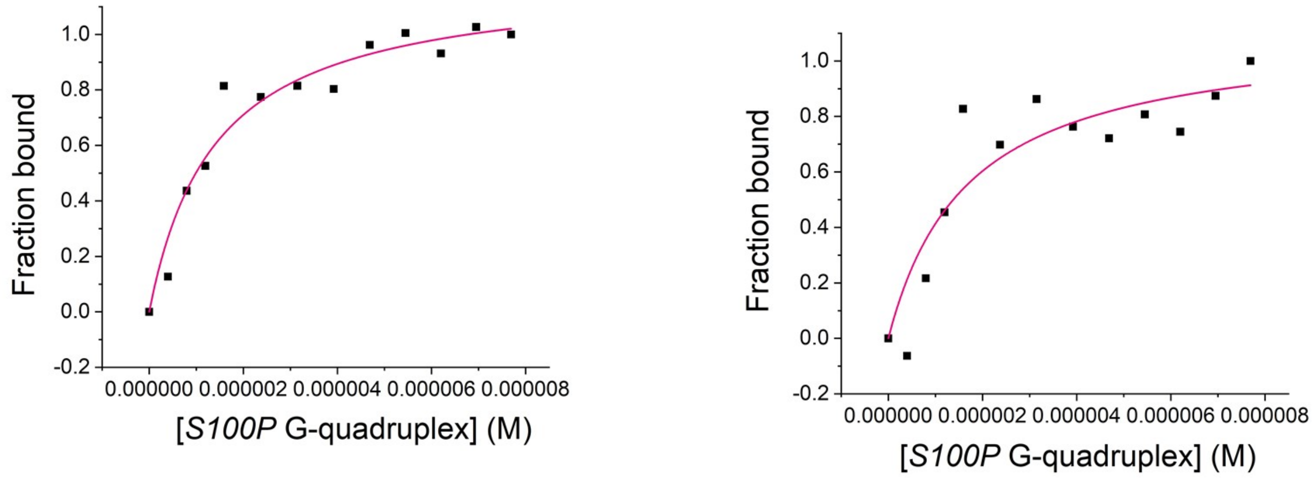

Figure S13. UV titration of QN-302 with *S100P* G-quadruplex. Fraction bound was calculated based on the change in the maximum absorbance after every addition of the DNA (method 2).

Table S2. Dissociation constants (k_d) and stoichiometry (n) of the *S100P* i-motif and G-quadruplex with the QN-302 ligand as determined by equations 1 and 2 by calculating the fraction bound based on methods 1 and 2. Values are average of two repeats.

	***S100P* i-motif**	***S100P* G-quadruplex**
**Method 1**		
k_d (μM)	11.7 ± 2.9	2.0 ± 0.3
n	1.7 ± 0.2	1.2 ± 0.0
**Method 2**		
k_d (μM)	10.8 ± 2.9	1.5 ± 0.2
n	1.6 ± 0.2	1.2 ± 0.1

References

(1) J. Redman, *Methods*, 2007, 43, **4**, 302-312.
